# A Novel Micromechanics-Model-Based Probabilistic Analysis Method for the Elastic Properties of Unidirectional CFRP Composites

**DOI:** 10.3390/ma15155090

**Published:** 2022-07-22

**Authors:** Meijuan Shan, Libin Zhao, Jinrui Ye

**Affiliations:** 1 Department of Mechanics, School of Physical Science and Engineering, Beijing Jiaotong University, Beijing 100044, China; mjshan@bjtu.edu.cn; 2 School of Mechanical Engineering, Hebei University of Technology, Tianjin 300401, China; 3 School of Astronautics, Beihang University, Beijing 100191, China; 4 Association for Science and Technology of Beihang University, Beijing 100191, China; yejinrui@buaa.edu.cn

**Keywords:** composites, elastic property, micromechanics theoretical model, probabilistic analysis method, Monte Carlo simulation

## Abstract

Considerable uncertainties in the mechanical properties of composites not only prevent them from having efficient applications but also threaten the safety and reliability of structures. In order to determine the uncertainty in the elastic properties of unidirectional CFRP composites, this paper develops a probabilistic analysis method based on a micromechanics theoretical model and the Monte Carlo simulation. Firstly, four commonly used theoretical models are investigated by calculating the deterministic elastic parameters of three unidirectional CFRP composites, which are compared with experimental outcomes. According to error analyses, the bridging model is the most brilliant one, with errors lower than 6%, which suggests that it can be used in probabilistic analyses. Furthermore, constituent parameters are regarded as normally distributed random variables, and the Monte Carlo simulation was used to obtain samplings based on the statistics of constituent parameters. The predicted probabilistic elastic parameters of the T800/X850 composite coincide with those from experiments, which verified the effectiveness of the developed probabilistic analysis method. According to the probabilistic analysis results, the statistics of the elastic parameters, the correlations between the elastic parameters, and their sensitivity to the constituent’s properties are determined. The moduli *E*_11_, *E*_22_, and *G*_12_ of the T800/X850 composite follow the lognormal distribution, namely, ln(*E*_11_)~*N*[5.15, 0.028^2^], ln(*E*_22_)~*N*[2.15, 0.024^2^], and ln(*G*_12_)~*N*[1.48, 0.038^2^], whereas its Poisson’s ratio, *v*_12_, obeys the normal distribution, namely, *v*_12_~*N*(0.33, 0.012^2^). Additionally, the correlation coefficients between *v*_12_ and *E*_11_/*E*_22_/*G*_12_ are small and thus can be ignored, whereas the correlation coefficients between any two of *E*_11_, *E*_22_, and *G*_12_ are larger than 0.5 and should be considered in the reliability analyses of composite structures. The developed probabilistic analysis method based on the bridging model and the Monte Carlo simulation is fast and reliable and can be used to efficiently evaluate the probabilistic properties of the elastic parameters of any unidirectional composite in the reliability design of structures in engineering practice.

## 1. Introduction

Carbon-fiber-reinforced polymer (CFRP) composites have been widely used in various engineering fields due to their high specific stiffness, high specific strength, outstanding designability, etc. Among the different types of CFRP composites, the unidirectional (UD) continuous carbon-fiber-reinforced resin matrix composite lamina, which is the basic building block of multidirectional laminates, is the most popular one that has been greatly developed until now. However, severe uncertainty exists in the mechanical properties of the composite lamina because of the inherently scattered mechanical properties of the constituent, the misaligned fiber distribution, the uncertain volume fraction of the constituent, etc. [1,2]. Generally, in order to ensure the safety and reliability of composite structures, large safety factors are used in traditional deterministic design [3]. This inefficient and uneconomical design leads to overweight structures. In other words, the superiority of advanced composite materials in weight savings is greatly reduced.

**Figure 1:**
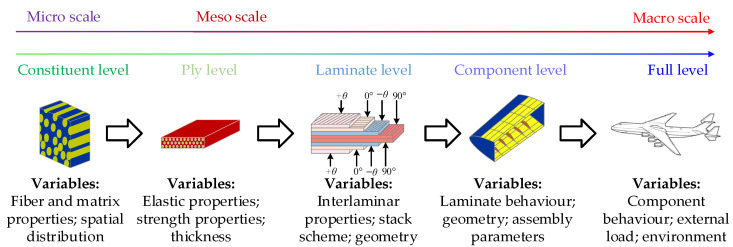
The transfer of uncertain mechanical properties from the constituent level to the full vehicle level.

In order to give full play to the potential of composites and obtain an efficient design of composite structures under the premise that high structural reliability and safety are guaranteed, probabilistic design methodologies have been developed and gradually applied to the design of engineering structures [4,5]. Multiscale methodologies have been developed to simulate the probabilistic mechanical properties of composite structures because of the uncertainty in the mechanical properties’ transfers from the micro-level to the structural level [6,7,8], as presented in Figure 1. Therefore, in probabilistic analyses of complex composite structures, the determination of the probabilistic mechanical properties of composite laminae is of great importance [9,10,11,12].

Up till now, the probabilistic mechanical properties of composite laminae have been investigated by two methodologies, which are the macro-level-started experimental method and the micro-level-started theoretical or numerical method. The former determines the statistics of mechanical properties based on numerous experimental data and probability distribution models [13,14,15,16,17,18]. For example, Jeong and Shenoi [14] obtained thirty-five experimental values of each mechanical parameter of composite laminae and determined their statistical characteristics by assuming that they follow the normal or Weibull distribution. However, the probability distribution type of the mechanical properties of composite laminae is found to influence the probabilistic strength of composite structures [15]. Furthermore, the large quantity of specimens required in this method indicates the high cost and long time period. To solve this problem, Sepahvand and Marburg [18] proposed a method for the optimal estimation of uncertain elastic parameters of composites from limited experimental modal data. However, correlations between the material properties still cannot be determined from this method. Therefore, the following question arises: will the statistical correlations between the material properties affect the mechanical reliability of composite structures? Shaw et al. [19] and Smarslok et al. [20] indicated that correlations between the lamina’s mechanical properties are significant for the reliability estimates of composite structures and that neglecting the correlations can lead to an inefficient or unsafe design. Zhang et al. [21] found that correlations between the lamina’s stiffness significantly affect the reliability of composite laminates, while the effect of correlations between the lamina’s strength is minimal.

In contrast, the micro-level-started theoretical or numerical method can not only determine the statistics of the material parameters but also obtain the correlations between these parameters. This method employs a micromechanics numerical model or theoretical model to calculate the probabilistic mechanical properties of the composite lamina while considering the randomness of the constituent’s properties. Lee et al. [22] and Mustafa et al. [23,24] combined micromechanics finite element models with statistical models of the constituents to determine the random mechanical properties of composite laminae. Jin et al. [25] used the micromechanics finite element, accompanied by a Monte Carlo simulation, to perform a probabilistic analysis of a plain weave carbon/epoxy composite. Bhattacharyya et al. [26] proposed a new micromechanics finite element model to achieve computational efficiency. Pitchai et al. [27] developed a homogenization technique based on the variational asymptotic method, coupled with the Monte Carlo method, to carry out an uncertainty and sensitivity analysis of the effective properties of unidirectional composites. Compared with the micromechanics finite element or other numerical models, micromechanics theoretical models are investigated more extensively, and advanced models have been developed in recent years. For example, Lezgy-Nazargah [28] proposed a micromechanics theoretical model based on iso-field assumptions to compute the effective coupled thermo-electro-elastic material properties of MFCs. In later studies, Lezgy-Nazargah and Eskandari-Naddaf [29] extended this model to estimate the material coefficients of three-phase piezoelectric structural fiber composites. The accuracy of the model was validated via comparison with the results of the rule of the mixture and a finite element model. Therefore, more researchers have used micromechanics theoretical models, including the rule of mixture [30], the model proposed by Halpin et al. [31], the model presented by Chamis et al. [32], and the bridging model proposed by Huang [33], to conduct probabilistic analyses of the mechanical properties of composite laminae [19,20,21,22,34,35]. As is well-known, the accuracy of a micromechanics theoretical model is the premise for obtaining the reliable probabilistic mechanical properties of composite laminae. However, little comparison has ever been made between micromechanics theoretical models to provide evidence for the selection of a model, which makes the existing probabilistic analysis method unsound.

To solve the above problem, this paper developed a novel probabilistic analysis method for the elastic properties of unidirectional CFRP composites on the basis of comprehensive comparisons being drawn between four commonly used micromechanics theoretical models. Firstly, the four models were employed to predict the deterministic elastic parameters of three unidirectional CFRP composites, and a detailed error analysis was conducted by comparing the predicted results with the experimental outcomes. Furthermore, a novel probabilistic analysis method that combines the bridging model, which was screened out of the four models, with the Monte Carlo simulation (MCS), was proposed. Basic constituent parameters, including the elastic parameters of the fiber and matrix as well as the volume fraction of the fiber, were regarded as random variables, and corresponding statistical models were established. The probabilistic elastic parameters obtained from the proposed method were compared with experimental outcomes to provide verification. Finally, the statistics of the elastic parameters, the correlations between the elastic parameters, and their sensitivity to the constituent’s properties were analyzed.

## 2. Methodology

The uncertainty in the mechanical properties of composites is caused by the uncertainty in the mechanical properties of their constituents. When random variables are used to describe the uncertainty in the mechanical properties of the constituent, the randomness in the mechanical properties of composites can be obtained. Therefore, a novel probabilistic analysis method that integrates a micromechanics theoretical model with the Monte Carlo simulation was developed to predict the random elastic parameters of unidirectional composites. The theoretical micromechanics model was used to calculate the elastic parameters, and the Monte Carlo simulation was utilized to obtain samplings of the constituent parameters based on statistics of the constituent parameters. Compared with the micromechanics finite element model or other numerical-model-based probabilistic analyses, the developed method based on the theoretical model is fast and reliable, and it can be used to efficiently evaluate the probabilistic properties of the elastic parameters of any unidirectional composite in the reliability design of complex structures in engineering practice.

**Figure 2:**
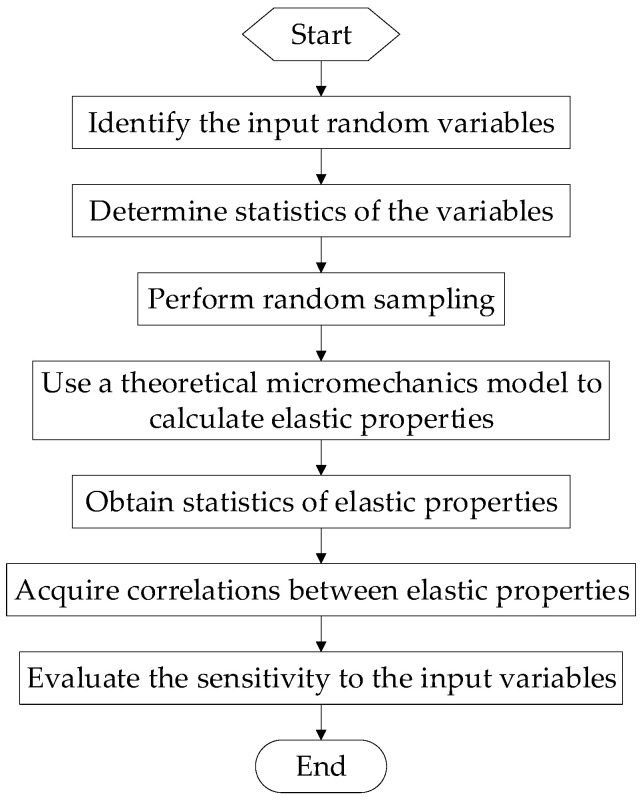
Flowchart for evaluating the probabilistic elastic properties of unidirectional composites.

The flowchart of the proposed method is shown in Figure 2, and the main steps are described as follows:

Step 1: Identify the input random variables, namely, the material properties of the constituent.
Step 2: Determine the statistics of the input random variables, including the probability distribution type and the distribution parameters.
Step 3: Perform random sampling using the Monte Carlo simulation.
Step 4: Use an accurate theoretical micromechanics model to calculate the elastic properties.
Step 5: Obtain the statistics of the elastic properties based on the output data.
Step 6: Acquire the correlations between the elastic properties.
Step 7: Evaluate the sensitivity of the elastic properties to the input constituent’s properties.

## 3. Comparisons of Micromechanics Models

Four commonly used micromechanics theoretical models were introduced and were utilized to calculate the elastic properties of three typical unidirectional CFRP composites. Detailed error analyses were conducted by comparing the calculated results with the experimental outcomes to screen out the most accurate one, which can be used in probabilistic analyses.

### 3.1. Descriptions of the Micromechanics Models

**Figure 3:**
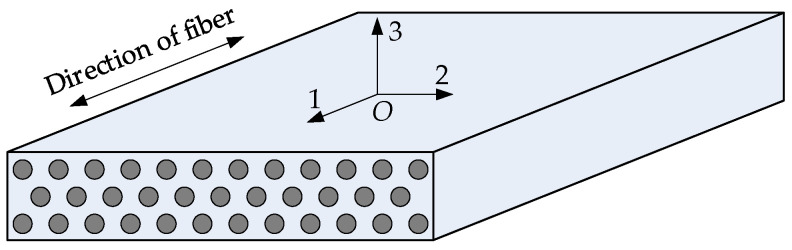
Schematic diagram of the unidirectional carbon-fiber-reinforced composite lamina.

The unidirectional continuous carbon-fiber-reinforced resin matrix composite lamina is the fundamental form of laminated composite structures. In the UD CFRP lamina, the carbon fibers are arranged in the same direction, as shown in Figure 3. As is well-known, the UD CFRP lamina is transversely isotropic because the carbon fiber is transversely isotropic and the resin matrix is isotropic. The material principal coordinate system *O*-123 of the lamina is also illustrated in Figure 3. The directions of the three principal axes of the lamina coincide with those of the carbon fibers.

The micromechanics theoretical models are established based on the assumptions that: (1) the fiber is uniformly distributed in the matrix; (2) the fiber and matrix are connected directly without relative sliding; (3) the volume fraction of the void is very small and thus can be ignored. Four micromechanics theoretical models that are widely used to predict the elastic constants of the UD CFRP composite lamina are introduced and investigated in this paper.

- Rule of mixture (numbered as Model I).

Among the micromechanics models, the rule of mixture [30] is the simplest one that has been used for a long time. The equations are the following:

(1) $$\begin{array}{l} E_{11}=V_{f}E_{11}^{f}+V_{m}E^{m}\\ v_{12}=V_{f}\nu_{12}^{f}+V_{m}\nu^{m}\\ E_{22}=\frac{E^{m}}{1-V_{f}\left(1-E^{m}/E_{22}^{f}\right)}\\ G_{12}=\frac{G^{m}}{1-V_{f}\left(1-G^{m}/G_{12}^{f}\right)} \end{array}$$

where $E_{11}^{f}$, $E_{22}^{f}$, $G_{12}^{f}$, and $\nu_{12}^{f}$ are the longitudinal elastic modulus, the transverse elastic modulus, the longitudinal transverse shear modulus, and the longitudinal transverse Poisson’s ratio of the fiber, respectively. $E^{m}$, $G^{m}$, and $\nu^{m}$ are the elastic modulus, the shear modulus, and the Poisson’s ratio of the matrix, respectively, among which the shear modulus is calculated by $G^{m}=E^{m}/2\left(1+\nu^{m}\right)$. $V_{f}$ and $V_{m}$ are the volume fractions of the fiber and the matrix, which satisfy $V_{f}+V_{m}=1$ because the volume fraction of the void is ignored.

- Chamis model (numbered as Model II).

Chamis [32] obtained a new model by simplifying the model proposed by Hopkins and Chamis [36]. The equations are expressed as follows:

(2) $$\begin{array}{l} E_{11}=V_{f}E_{11}^{f}+V_{m}E^{m}\\ v_{12}=V_{f}v_{12}^{f}+V_{m}v^{m}\\ E_{22}=\frac{E^{m}}{1-\sqrt{V_{f}}\left(1-E^{m}/E_{22}^{f}\right)}\\ G_{12}=\frac{G^{m}}{1-\sqrt{V_{f}}\left(1-G^{m}/G_{12}^{f}\right)} \end{array}$$

It can be seen from Equation (2) that the equations for *E*_22_ and *G*_12_ are similar to those in the rule of mixture because only the $V_{f}$ is replaced with $\sqrt{V_{f}}$.

- Halpin–Tsai model (numbered as Model III).

Halpin et al. [31] summarized the Halpin–Tsai equations in detail, in which the equations for the elastic parameters are the following:

(3) $$\begin{array}{l} E_{11}=V_{f}E_{11}^{f}+V_{m}E^{m}\\ v_{12}=V_{f}v_{12}^{f}+V_{m}v^{m}\\ E_{22}=E^{m}\left(\frac{1+2\alpha V_{f}}{1-\alpha V_{f}}\right)\begin{array}{cc} & \alpha =\frac{\left(E_{22}^{f}/E^{m}\right)-1}{\left(E_{22}^{f}/E^{m}\right)+2} \end{array}\\ G_{12}=G^{m}\left(\frac{1+\beta V_{f}}{1-\beta V_{f}}\right)\begin{array}{cc} & \beta =\frac{\left(G_{12}^{f}/G^{m}\right)-1}{\left(G_{12}^{f}/G^{m}\right)+1} \end{array} \end{array}$$

- Bridging model (numbered as Model IV).

Huang established the bridging model [33], which can predict both the elastic and the strength parameters of unidirectional composites. The equations for the elastic parameters are the following:

(4) $$\begin{array}{l} E_{11}=V_{f}E_{11}^{f}+V_{m}E^{m}\\ v_{12}=V_{f}v_{12}^{f}+V_{m}v^{m}\\ E_{22}=\frac{\left(V_{f}+V_{m}a_{11}\right)\left(V_{f}+V_{m}a_{22}\right)}{\left(V_{f}+V_{m}a_{11}\right)\left(V_{f}S_{22}^{f}+a_{22}V_{m}S_{22}^{m}\right)+V_{f}V_{m}\left(S_{21}^{m}-S_{21}^{f}\right)a_{12}}\\ G_{12}=G^{m}\frac{\left(G_{12}^{f}+G^{m}\right)+V_{f}\left(G_{12}^{f}-G^{m}\right)}{\left(G_{12}^{f}+G^{m}\right)-V_{f}\left(G_{12}^{f}-G^{m}\right)} \end{array}$$

where $a_{11}=E^{m}/E_{11}^{f}$, $a_{22}=0.5\left(1+E^{m}/E_{22}^{f}\right)$, $a_{12}=\left(S_{12}^{f}-S_{12}^{m}\right)\left(a_{11}-a_{22}\right)/\left(S_{11}^{f}-S_{11}^{m}\right)$, $S_{11}^{f}=1/E_{11}^{f}$, $S_{22}^{f}=1/E_{22}^{f}$, $S_{12}^{f}=S_{21}^{f}=-\nu_{12}^{f}/E_{11}^{f}$, $S_{11}^{m}=S_{22}^{m}=1/E^{m}$, and $S_{12}^{m}=S_{21}^{m}=-\nu^{m}/E^{m}$. It can be found from a derivation that the equation for *G*_12_ is the same as that in the Halpin–Tsai model.

As discussed above, the equations for *E*_11_ and *v*_12_ are the same in the four micromechanics models, whereas the equations for *E*_22_ and *G*_12_ are different. When the four basic elastic parameters are obtained, the other elastic parameters of the unidirectional composite lamina can be determined according to the transversely isotropic assumption, except that *v*_23_ is calculated by the equation of Christensen [37]. The equations are formulated as follows:

(5) $$\begin{array}{l} E_{33}=E_{22}\;\\ G_{13}=G_{12}\;\\ \nu_{13}=\nu_{12}\\ \nu_{23}=\nu_{12}\left(1-\nu_{12}E_{22}/E_{11}\right)/\left(1-\nu_{12}\right)\\ G_{23}=E_{22}/2\left(1+\nu_{23}\right) \end{array}$$

### 3.2. Results and Analyses

The four micromechanics theoretical models were utilized to predict the elastic parameters of three typical unidirectional CFRP composites. As shown in Table 1, the three unidirectional carbon-fiber-reinforced epoxy resin matrix composites chosen are all extensively applied in the aerospace field as well as other engineering fields. For example, the T800/X850 composite, fabricated from T800 carbon fiber and CYCOM X850 epoxy resin, is widely utilized in the primary structures of large aircraft. The fiber volume fractions and the elastic properties of the constituents of the three composites are listed in Table 1.

**Figure 4:**
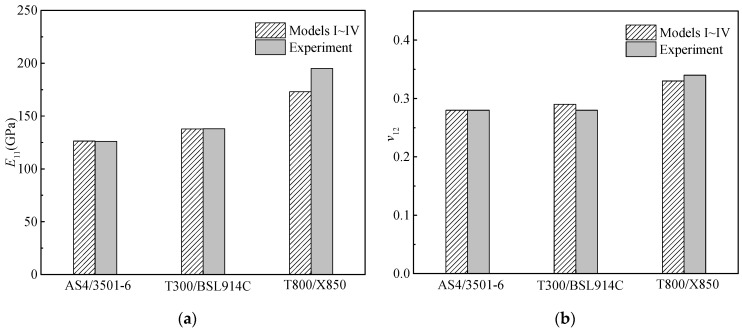

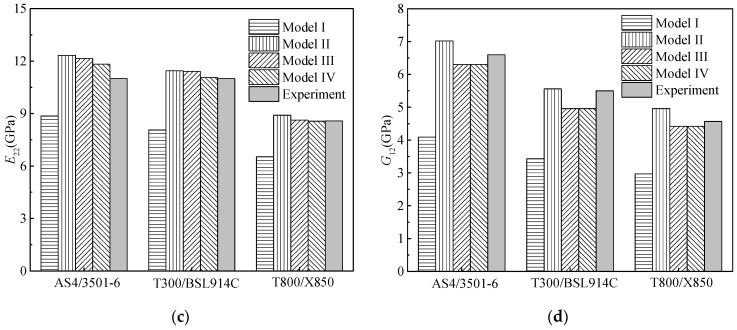
Comparisons between the calculated elastic properties and the experimental values of the three composites. (**a**) *E*_11_; (**b**) *v*_12_; (**c**) *E*_22_; (**d**) *G*_12_.

The elastic properties of the three composites calculated by the four micromechanics models as well as the experimental results are presented in Figure 4. The experimental results of the AS4/3501−6 and T300/BSL914C composites are from [41]. The experimental results of the T800/X850 composite are the means of fifteen test values provided by the manufacturer. It is worth noting that the experimental data of the three composites are obtained by using the lamina with a regular pattern of fibers, which indicates that the fiber can be considered to be uniformly distributed in the matrix. As illustrated in Figure 4, small differences are observed between the theoretical and experimental values of *E*_11_ and *v*_12_. The theoretical *E*_22_ and *G*_12_ obtained from Model I differ greatly from the experimental values, while those obtained from Models II to IV show relatively small differences from the experimental values. Model I, namely, the rule of mixture, is accurate in calculating *E*_11_ and *v*_12_, but seriously underestimates *E*_22_ and *G*_12_. This is consistent with the findings in [22,23,42], which pointed out that when using the rule of mixture, the values for *E*_22_ and *G*_12_ showed a large difference. Models II–IV modified the formulae of *E*_22_ and *G*_12_ and therefore can evaluate the values of *E*_22_ and *G*_12_ more accurately. This conforms to the findings in [19,20,21,22,23,34]. For example, Model II is used to obtain the elastic parameters of unidirectional composites in [20,35]. Model III is used to validate the results of finite element models in [22,23], which suggests the accuracy of Model III. Furthermore, Model IV is widely used in obtaining the random elastic parameters of unidirectional composites when the multiscale uncertainty of laminated structures is investigated [19,21], which demonstrates the precision of Model IV.

**Figure 5:**
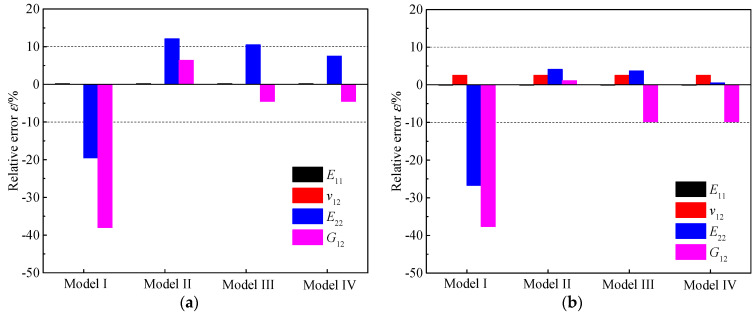

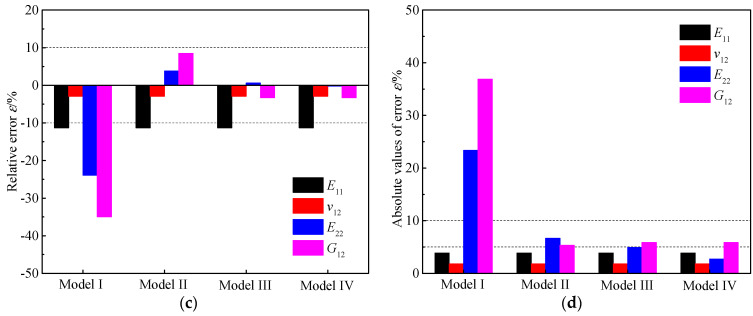
Relative error between the theoretical and experimental elastic parameters of the three composites. (**a**) AS4/3501−6; (**b**) T300/BSL914C; (**c**) T800/X850; (**d**) Mean.

Furthermore, the relative errors, *ε*, obtained by comparing the theoretical values with the experimental values are depicted in Figure 5a–c. Since the equations for *E*_11_ and *v*_12_ are the same in all four models, their theoretical values are equal. Additionally, the errors of *E*_11_ and *v*_12_ are very small, except that the *E*_11_ of the T800/X850 composite has an error greater than −10%. The *E*_22_ obtained from Model I is remarkably smaller than the test values, with errors up to −27%, while those obtained from Models II to IV are larger than the experimental values, with decreasing errors in sequence, which are below 12%. The *G*_12_ obtained from Model I is also far less than the test values, with errors higher than −35%; however, those obtained from Model II are larger than the test values, with errors below 8.5%, and those acquired from Models III to IV are smaller than the test values, with errors below −10%. To conclude, for the three composites, the errors of Model I are unacceptably large, and the errors of Models II–IV remain approximately below 10%. This is in accordance with the findings in [19,20,21,22,23,34,35,42], which demonstrated that Model I shows large differences from the experimental or numerical results, whereas Models II–IV differ slightly from the experimental or numerical results.

As discussed above, Models II–IV show similar high precision in calculating the elastic parameters of unidirectional composites. In order to pick out the most accurate one, the mean of the absolute values of the errors, $\varepsilon_{\mathrm{mean}}$, is calculated by $\varepsilon_{\mathrm{mean}}=\left(\left|\varepsilon_{\mathrm{AS}4}\right|+\left|\varepsilon_{T300}\right|+\left|\varepsilon_{T800}\right|\right)/3$. According to Figure 5d, the *E*_11_ and *v*_12_ obtained from the four models show small errors of 3.87% and 1.80%, respectively. The *E*_22_ and *G*_12_ determined by Model I have remarkable errors, higher than 23%, whereas those acquired from Models II–IV have small errors, lower than 7%. Concerning Models II–IV, the errors of *E*_22_ decrease in sequence, and the errors of *G*_12_ are similar. Therefore, Model IV, namely, the bridging micromechanics model, is the most brilliant one, which can obtain relatively accurate *E*_22_ and *G*_12_, with errors of 2.73% and 5.87%, respectively. For this reason, the bridging model is widely used in mechanical analyses of unidirectional structures [19,21,43,44,45]. Therefore, the bridging model was used in the probabilistic analysis of the elastic parameters of unidirectional composites.

## 4. Predictions of Random Elastic Properties

In order to verify the proposed method, the probabilistic elastic properties of the T800/X850 composite were predicted and compared with the experimental outcomes. Furthermore, the statistics of the elastic parameters, the correlations between these elastic parameters, and their sensitivity to the constituent’s properties were analyzed.

### 4.1. Validation

All of the involved constituent properties of the T800/X850 composite are considered random variables. There are seven mutually independent random variables. The statistics of these random variables are presented in Table 2. The constituent’s properties are assumed to follow the normal distribution. The coefficients of variance (COVs) of the moduli and fiber volume fraction are 0.02, and the COVs of the Poisson’s ratios are 0.05 [19,21,23,34,46]. Furthermore, 10^5^ samples of the random variables are obtained using the ziggurat method in the MATLAB^®^ software. According to the bridging micromechanics model, 10^5^ samples of the four elastic parameters are acquired.

**Figure 6:**
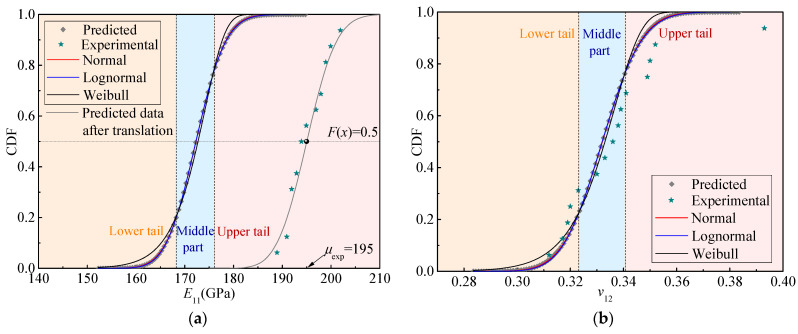

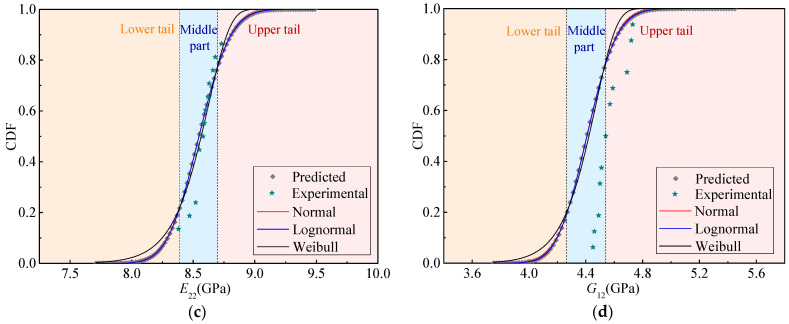
Cumulative distribution functions (CDFs) of the elastic properties of the T800/X850 composite. (**a**) *E*_11_; (**b**) *v*_12_; (**c**) *E*_22_; (**d**) *G*_12_.

Figure 6 represents the calculated cumulative distribution probabilities of the four elastic parameters. The cumulative probabilities obtained from the experiments, which are also shown in Figure 6, are calculated by the following [15]:

(6) $$P_{\exp}=m/\left(n+1\right)$$

where *m* denotes the sequence number of the value when fifteen test values of each parameter are arranged in ascending order, and *n* is the number of the specimens. It can be seen from Figure 6 that the predicted cumulative probabilities of *v*_12_, *E*_22_, and *G*_12_ agree well with the experimental outcomes, whereas the predicted cumulative probability of *E*_11_ shows a substantial discrepancy from that of the experiments. As shown in Figure 6a, the predicted data are horizontally translated until arriving at the point determined by the experimental mean and the cumulative probability, 0.5. Then, the predicted result coincides well with the experimental outcome. It is in fact the case that the remarkable deviation happens because the predicted *E*_11_ in the deterministic analysis (approximately corresponding to the mean of *E*_11_ in the probabilistic analysis) is remarkably smaller than the test values. This provides evidence that the proposed method can efficiently evaluate the probabilistic properties of the elastic parameters of the composites with adequate accuracy under the premise that the deterministic elastic parameters can be accurately predicted.

### 4.2. Statistics of Elastic Parameters

In order to determine the statistics of the four elastic parameters, the cumulative distribution functions (CDFs) of the normal, lognormal, and Weibull distributions were employed to fit the cumulative distribution probabilities calculated from the proposed method. The fitting equations are the following:

(7) $$\begin{array}{l} F(x)|{}_{\mathrm{Normal}}=\frac{1}{2}\left[1+\mathrm{erf}\left(\frac{x-\mu }{\sqrt{2}\sigma }\right)\right]\\ F(x)|{}_{\mathrm{Lognormal}}=\frac{1}{2}\left[1+\mathrm{erf}\left(\frac{\ln x-\mu }{\sqrt{2}\sigma }\right)\right]\\ F(x)|{}_{\mathrm{Weibull}}=1-\exp\left[-{\left(\frac{x}{\lambda }\right)}^{\kappa }\right] \end{array}$$

where the error function is $\mathrm{erf}(x)=\frac{2}{\sqrt{\pi }}\int_{0}^{x}e^{-t^{2}}dt$. The fitting curves of the four elastic parameters are shown in Figure 6. The CDFs fitted by the normal distribution are almost consistent with those fitted by the lognormal distribution, and both of them agree well with the predicted data, which demonstrates that these two distributions are almost equivalent for describing the statistics of the elastic parameters. The CDFs fitted by the Weibull distribution deviate slightly from the predicted data at the middle part but exhibit notable differences from the predicted data in the lower and upper tails. Consequently, the four random elastic parameters are inclined to obey the normal or lognormal distribution.

Moreover, Table 3 presents the fitted distribution parameters as well as the adjusted coefficient of determination, *R*^2^, which denotes the goodness of a fit. An adjusted *R*^2^ close to 1.0 suggests that the fit is the best one of the three distributions. The adjusted *R*^2^ values of the Weibull distribution are smaller than those of the normal and lognormal distributions. According to the comparisons made between the adjusted *R*^2^ values of the normal and lognormal distributions, *E*_11_, *E*_22_, and *G*_12_ tend to follow the lognormal distribution, while *v*_12_ inclines to obey the normal distribution.

**Figure 7:**
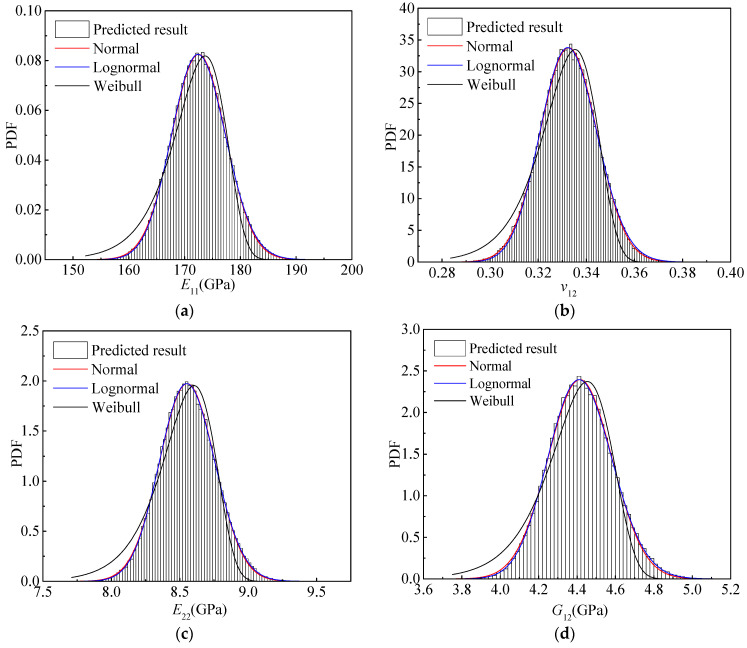
Histograms and PDFs of the elastic properties of the T800/X850 composite. (**a**) *E*_11_; (**b**) *v*_12_; (**c**) *E*_22_; (**d**) *G*_12_.

Furthermore, the histograms of the four elastic parameters are shown in Figure 7. The probability density functions (PDFs) of the normal, lognormal, and Weibull distributions are applied to fit the histograms of the four parameters, as presented in Figure 7. The PDFs of the Weibull distribution show substantial discrepancies from the histograms. Although the PDFs of both the normal and lognormal distributions coincide with the histograms, subtle differences can be observed between them. As for *E*_11_, *E*_22_, and *G*_12_, the PDFs of the lognormal distribution agree better with the histograms than those of the normal distribution. Additionally, an opposite tendency appears for *v*_12_.

Based on the above discussions, *E*_11_, *E*_22,_ and *G*_12_ obey the lognormal distribution, whereas *v*_12_ follows the normal distribution.

### 4.3. Correlation Analysis

**Figure 8:**
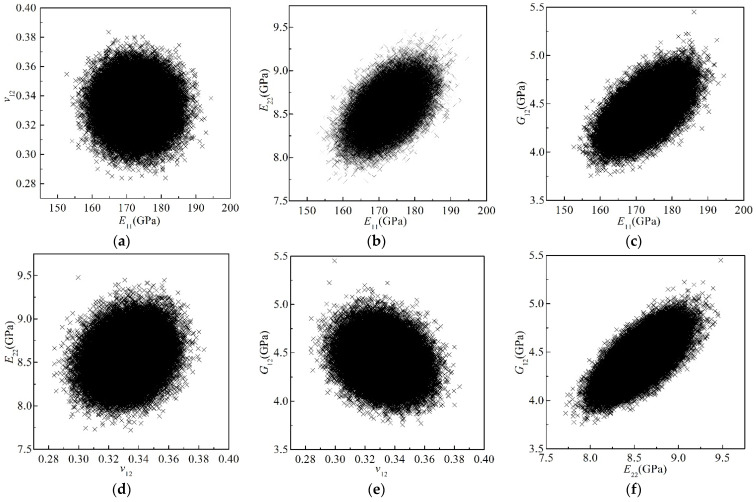
Scatter plot of the four elastic parameters of the T800/X850 composite. (**a**) *E*_11_ vs. *v*_12_; (**b**) *E*_11_ vs. *E*_22_; (**c**) *E*_11_ vs. *G*_12_; (**d**) *v*_12_ vs. *E*_22_; (**e**) *v*_12_ vs. *G*_12_; (**f**) *E*_22_ vs. *G*_12_.

The scatter plots of the calculated 10^5^ samples of the four elastic parameters are shown in Figure 8. The distribution of the data reflects the correlations between the two parameters. The circle-like distributions in Figure 8a,d,e suggest weak correlations between *v*_12_ and the other three elastic parameters. The ellipse-like distributions in Figure 8b,c,f denote strong correlations between any two of the three moduli.

**Figure 9:**
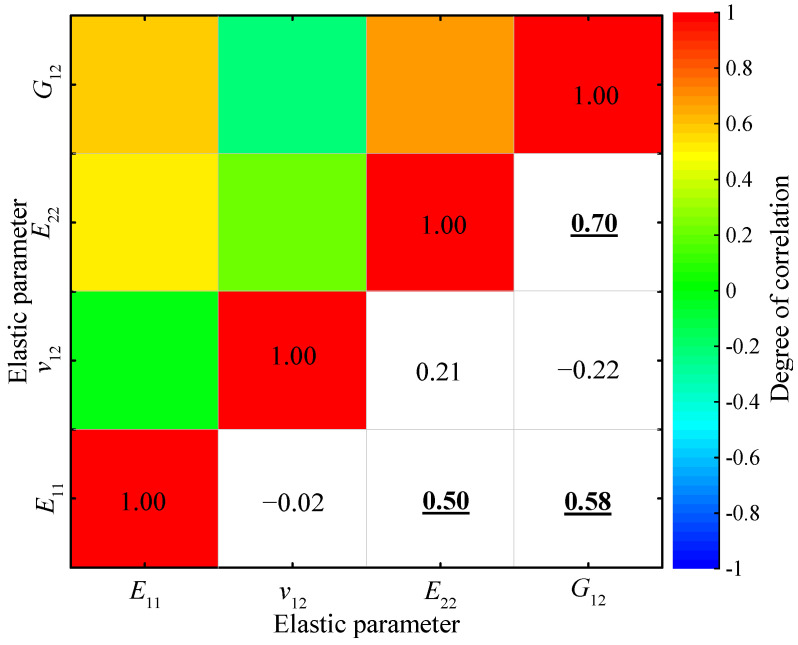
Correlation coefficients between the four elastic properties of the T800/X850 composite.

Further, the Pearson correlation coefficient was employed to quantitatively represent the correlations between the elastic parameters. The Pearson correlation coefficient is calculated using Equation (8). The value of the correlation coefficient is in the range of [−1.0, 1.0]. When it is close to 0, the two variables, *X* and *Y*, are not or weakly relevant. The closer it gets to −1.0 or 1.0, the stronger the correlation is. A value in the range of (0, 1] means a positive correlation, whereas a value in the range of [−1, 0) suggests a negative correlation, as follows:

(8) $$\rho \left(X,Y\right)=\frac{\sum XY-\frac{\sum X\sum Y}{N}}{\sqrt{\left(\sum X^{2}-\frac{{\left(\sum X\right)}^{2}}{N}\right)\left(\sum Y^{2}-\frac{{\left(\sum Y\right)}^{2}}{N}\right)}}$$

where *X* and *Y* are random variables, whereas *N* is the sampling number of the variable. As shown in Figure 9, the correlation coefficients between *v*_12_ and the other three elastic parameters are small and therefore can be ignored. However, the correlation coefficients between *E*_11_ and *E*_22_, between *E*_11_ and *G*_12_, and between *E*_22_ and *G*_12_ are larger than 0.5, indicating that the correlations between these three elastic parameters should be considered in reliability analyses of the composite structures.

### 4.4. Sensitivity Analysis

**Figure 10:**
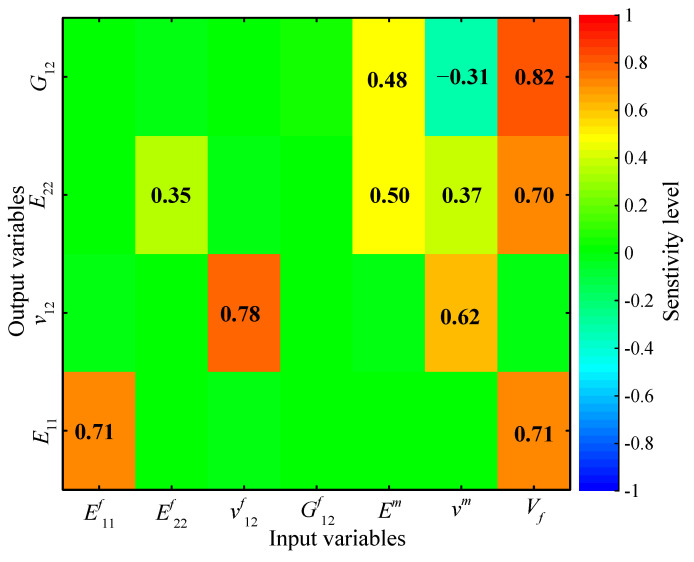
Correlation coefficients between the elastic properties and the constituent’s properties of the T800/X850 composite.

The probabilistic sensitivities were evaluated according to the Pearson correlation coefficients between a particular output parameter and all of the input random variables. The analysis results are illustrated in Figure 10, where the absolute values larger than 0.3 are marked. The longitudinal elastic modulus, *E*_11_, has strong relations with $E_{11}^{f}$ and $V_{f}$. The transverse elastic modulus, *E*_22_, depends on $V_{f}$, $E^{m}$, $v^{m}$, and $E_{22}^{f}$ in sequence. The shear modulus, *G*_12_, is highly related to $V_{f}$, $E^{m}$, and $v^{m}$. The Poisson’s ratio, *v*_12_, mainly relates to $v_{12}^{f}$ and $v^{m}$.

## 5. Conclusions

A novel probabilistic analysis method was proposed to determine the uncertainty in the elastic properties of unidirectional CFRP composites. The method integrates a micromechanics theoretical model with the Monte Carlo simulation. Firstly, comprehensive comparisons between four commonly used theoretical models were made by comparing the calculated deterministic elastic parameters of three unidirectional CFRP composites with experimental outcomes in the literature. According to detailed error analyses, the bridging model is the most accurate one, with errors lower than 6%, which suggests that it can be used in probabilistic analyses. Moreover, constituent parameters, including the elastic parameters of the fiber and the matrix as well as the volume fraction of fiber, were regarded as normally distributed random variables, and the Monte Carlo simulation was used to obtain samplings according to statistics of the constituent parameters. The proposed method was used to predict the random elastic parameters of the T800/X850 composite, and the predicted results were compared with the experimental outcomes to provide validation. Additionally, correlations between the elastic parameters and their sensitivity to the constituent’s properties were also determined. The following conclusions can be drawn: (1) Among the four micromechanics theoretical models, the bridging model is proven to be the best one that can accurately predict the elastic properties of unidirectional CFRP composites. (2) The proposed probabilistic analysis method, based on the bridging model and the Monte Carlo simulation, can efficiently evaluate the random elastic parameters of unidirectional CFRP composites with adequate accuracy. (3) The moduli *E*_11_, *E*_22_, and *G*_12_ of the T800/X850 composite follow the lognormal distribution, while its Poisson’s ratio, *v*_12_, obeys the normal distribution. Namely, ln(*E*_11_)~*N*[5.15, 0.028^2^], ln(*E*_22_)~*N*[2.15, 0.024^2^], ln(*G*_12_)~*N*[1.48, 0.038^2^], and *v*_12~_*N*(0.33, 0.012^2^). (4) The correlation coefficients between *v*_12_ and *E*_11_/*E*_22_/*G*_12_ are small and thus can be ignored, whereas the correlation coefficients between any two of *E*_11_, *E*_22_, and *G*_12_ are larger than 0.5 and should be considered in multiscale uncertainty analyses of composite structures. (5) The longitudinal elastic modulus, *E*_11_, has strong relations with $E_{11}^{f}$ and $V_{f}$. The transverse elastic modulus, *E*_22_, depends on $V_{f}$, $E^{m}$, $v^{m}$, and $E_{22}^{f}$ in sequence. The shear modulus, *G*_12_, is highly related to $V_{f}$, $E^{m}$, and $v^{m}$. The Poisson’s ratio, *v*_12_, mainly relates to $v_{12}^{f}$ and $v^{m}$.

In the future, to obtain an efficient design of composite structures, multiscale uncertainty design and analysis of complex composite structures will be widely applied. Additionally, the determination of uncertainty in the mechanical properties of a composite lamina is of great importance because the uncertainty in the mechanical properties transfers from the micro-level to the structural level. This paper provides a fast and reliable probabilistic analysis method for the uncertainty evaluation of the elastic parameters of any unidirectional composite. The method is based on the theoretical bridging model and the Monte Carlo simulation, and it can be easily packaged and embedded in software via a programming language, which suggests its great potential in applications in engineering practice.

## Figures and Tables

**Table 1:** Material properties of the constituent of the three unidirectional composites.

Title	Fiber Volume Fraction	Elastic Properties of the Fiber				Elastic Properties of the Matrix	
	$V_{f}$	$E_{11}^{f}\;(\mathrm{GPa})$	$E_{22}^{f}\;(\mathrm{GPa})$	$G_{12}^{f}\;(\mathrm{GPa})$	$\nu_{12}^{f}$	$E^{m}\;(\mathrm{GPa})$	$\nu^{m}$
AS4/3501−6 [38]	0.60	207.5	25	95	0.240	4.5	0.34
T300/BSL914C [39]	0.60	227	25	28	0.245	4.0	0.35
T800/X850 [40]	0.58	295	17.1	40.9	0.32	3.52	0.35

**Table 2:** Statistics of the constituent’s properties of the T800/X850 composite.

Random Variable	Mean	COV	Distribution Type
$E_{11}^{f}$ (GPa)	295	0.02	Normal
$E_{22}^{f}$ (GPa)	17.1	0.02	Normal
$G_{12}^{f}$ (GPa)	40.9	0.02	Normal
$\nu_{12}^{f}$	0.32	0.05	Normal
$E^{m}$ (GPa)	3.52	0.02	Normal
$\nu^{m}$	0.35	0.05	Normal
$V_{f}$	0.58	0.02	Normal

**Table 3:** Statistical models of the elastic properties of the T800/X850 composite.

Random Variable	Normal Distribution			Lognormal Distribution			Weibull Distribution		
	*μ*	*σ*	Adj. *R*^2^	*μ*	*σ*	Adj. *R*^2^	*λ*	*κ*	Adj. *R*^2^
*E*_11_ (GPa)	172	4.83	0.99999	5.15	0.028	1.00000	174	42.10	0.99760
*v*_12_	0.33	0.012	1.00000	−1.10	0.036	0.99997	0.34	33.12	0.99781
*E*_22_ (GPa)	8.55	0.202	0.99998	2.15	0.024	1.00000	8.63	49.85	0.99736
*G*_12_ (GPa)	4.41	0.167	0.99996	1.48	0.038	1.00000	4.47	31.19	0.99771

## Data Availability

Data sharing is not applicable to this article.
